# Glycolipid transfer protein knockout disrupts vesicle trafficking to the plasma membrane

**DOI:** 10.1016/j.jbc.2023.104607

**Published:** 2023-03-15

**Authors:** Henrik Nurmi, Anders P.E. Backman, Josefin Halin, Max Lönnfors, Tomas Blom, Pia Roos-Mattjus, Peter Mattjus

**Affiliations:** 1 Department of Biochemistry, Faculty of Science and Engineering, Åbo Akademi University, Turku, Finland; 2 Department of Anatomy and Research Programs Unit, Faculty of Medicine, University of Helsinki, Helsinki, Finland

**Keywords:** endoplasmic reticulum (ER), Golgi, gene silencing, glycerosphingolipid, glycolipid, intracellular trafficking, lipid transport, VAP-A, VSVG, sphingolipid

## Abstract

The glycolipid transfer protein (GLTP) has been linked to many cellular processes aside from its best-known *in vitro* function as a lipid transport protein. It has been proposed to act as a sensor and regulator of glycosphingolipid homeostasis in cells. Furthermore, through its previously determined interaction with the endoplasmic reticulum membrane protein VAP-A (vesicle-associated membrane protein-associated protein A), GLTP may also be involved in facilitating vesicular transport in cells. In this study, we characterized the phenotype of CRISPR/Cas9-mediated GLTP KO HeLa cells. We showed that motility, three-dimensional growth, and cellular metabolism were all altered by GLTP knockout. Expression of a GLTP mutant incapable of binding VAP disrupted cell spheroid formation, indicating that the GLTP–VAP interaction is linked to cellular adhesion, cohesion, and three-dimensional growth. Most notably, we found evidence that GLTP, through its interaction with VAP-A, affects vesicular trafficking, marking the first cellular process discovered to be directly impacted by a change in GLTP expression.

The glycolipid transfer protein, GLTP is a cytosolic, evolutionarily highly conserved protein with a unique structural conformation (eight α-helices organized in two layers), unique enough that this type of structural conformation has been designated the “GLTP fold” (1, 2). GLTP is named for its first discovered function, facilitating the transport of glycosphingolipids between artificial lipid membranes *in vitro* (3, 4). Numerous homologs with similar functions have also been discovered, with GLTP recognized as the prototypical member of a superfamily (the GLTP superfamily) of structurally similar nonenzymatic lipid transfer proteins (2). The role of GLTP as a sensor and regulator of glycolipid homeostasis was proposed earlier (5) and, while GLTP displays broad specificity for its substrates, it has been demonstrated to differentiate between glycosphingolipids of different acyl chain lengths (6). This lends credence to the theory that GLTP, by virtue of its capability to distinguish between glycosphingolipid species, could act as a regulator of glycosphingolipid levels downstream of the glycosphingolipid synthesis and differentiation pathway. Further supporting this theory, our previous studies have discovered that variations in lipid levels in cells affect the expression levels of GLTP, and conversely, that GLTP expression levels affect the lipidomes of cells (7, 8).

While GLTP is a cytosolic protein, it possesses an FFAT structural motif (two phenylalanines in an acidic tract), suggesting that it is targeted to the surface of the endoplasmic reticulum, since proteins possessing an FFAT targeting motif are known to interact with vesicle-associated membrane protein-associated protein (VAPs) on the (endoplasmic reticulum) ER’s surface (9, 10, 11). Aside from GLTP, such proteins include other lipid transport proteins, such as the oxysterol-binding protein and its related proteins among others (12, 13). The interaction of these proteins with VAPs has been shown to affect vesicular transport between the ER and the plasma membrane (13, 14). Since GLTP has also been shown to interact with VAP *via* its FFAT domain (15) and may therefore also influence vesicle transport. Targeting to the surface of the ER also places GLTP near the early stages of lipid biosynthesis in the ER and we have shown that the action of GLTP binding a lipid substrate inhibits the interaction between GLTP and VAP-A (6). This potential regulatory mechanism could tie the interaction of GLTP with VAP to changes in glycolipid expression levels, meaning that GLTP, through its role in detecting and maintaining glycosphingolipid levels, could potentially affect vesicular trafficking in response to the needs of the cell as mediated by changes to the lipidome (6). It would also imply that changes to the lipid binding function or to the interaction of GLTP with VAP could potentially have deleterious effects on the regulation and function of the vesicular transport chains in the cell. In fact, two key structural components have been identified in GLTP that are of particular and specific interest in this matter: the FFAT domain, which facilitates interaction with VAP-A as described above, and tryptophan residue W96, which has been identified as central to the functioning of the glycosphingolipid binding site of the protein (16, 17). GLTP mutants that target these key components have been generated and since these mutants disable specific interactions and functions of GLTP, they are valuable tools for determining the roles of these functions in a cellular environment.

The aim of this study was to determine and characterize the overall *in vivo* significance of GLTP. We used CRISPR/Cas9 to generate a stable knockout of the gene that encodes for GLTP in HeLa cells. Three GLTP KO lines were generated (henceforth referred to as “GLTP KO cells” or “KO cells”) and the success of the gene knockout was validated *via* Western blot analysis with α-GLTP antibodies. As inferred from the success in generating viable KO cell lines, the absence of GLTP is not lethal to cells; thus, we could immediately conclude that GLTP is not strictly required for the continued survival of cells. The lipidomes of cells were shown to be affected by the gene knockout, as total cellular glycosphingolipid levels in the KO cells were observed to be lower than in corresponding WT cells (Cas9 GLTP control), further supporting the idea that GLTP acts as a regulator of glycosphingolipid levels in cells. Certain changes in cell behavior and functions were also observed to have been caused by knocking out GLTP production, such as changes in cell migration rates. GLTP knockout did not noticeably affect cell proliferation rates and the effects of knockout on spheroid formation were small. More notably, we found that GLTP knockout affected cellular metabolism, increasing the oxygen consumption rate (OCR) of the GLTP KO cells. We also used the KO cells to reintroduce WT GLTP into the cells as a rescue assay and to study the effects of structural GTLP mutations on cells and cellular processes by introducing these mutants into the KO cells *via* transient transfection. Finally, we found that the absence of GLTP indeed affected vesicular trafficking, since vesicle transport in GLTP KO cells appeared to be significantly disrupted compared to transport in WT HeLa cells. By studying the effects of structural GLTP mutations, we determined that the interaction between GLTP and VAP-A is indeed responsible for maintaining functioning of the vesicle transport chain in cells and that disabling this interaction in turn disrupts this transport chain.

## Results

### Knockout of the GLTP gene in HeLa cells

To generate a GLTP gene knockout in HeLa cells, we designed a target deletion in exon 1 using the CRISPR/Cas9 online predictor CRISPR design tool from Ensembl. CRISPR/Cas9-treated cells were clonally selected for GLTP KO and WT cells were selected for comparison and intron l of GLTP was sequenced. All the KO cell lines all had major deletions, insertions, and slips in intron 1 of GLTP, while the WT cell lines had a fully intact intron 1 (Fig. S1).

### Glycosphingolipid expression was altered in KO cells

**Figure 1:**
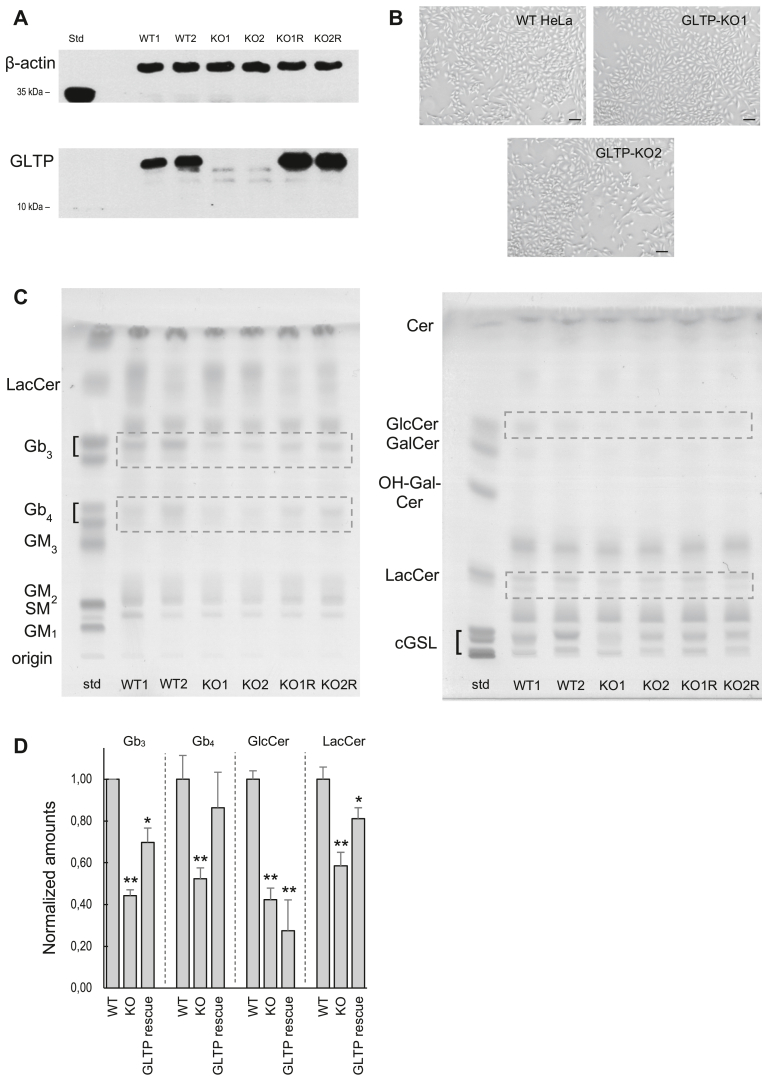
**GLTP knockout in HeLa cells lowers globoside, glucosylceramide, and lactosylceramide levels.***A*, Western blot analysis of GLTP expression in two WT (WT1 and WT2), two GLTP KO (KO1 and KO2) cell lines, and two GLTP KO rescue (KO1R and KO2R) cell types with anti-GLTP antibody. Beta-actin was used as a loading control. *B*, light microscopy images of WT GLTP, GLTP KO1, and GLTP KO2 HeLa cultures. The scale bars represent 200 μm. *C*, HPTLC analysis of glycosphingolipids in two WT (WT1 and WT2), two GLTP KO (KO1 and KO2), and two GLTP KO rescue (KO1R and KO2R) HeLa cell types. The plates were stained with carbohydrate-specific orcinol to stain the lipids. The *left-hand* HPTLC plate shows the globoside (Gb) separation from other GSLs using the 25:20:4:1 chloroform:methanol:HAc:H_2_O (v/v) solvent system. The *right-hand* HPTLC plate shows the separation of GlcCer, GalCer, and LacCer using the solvent system 10:2:4:2:1 chloroform:methanol:acetone:HAc:H_2_O (v/v). When GLTP was absent, the levels of GlcCer, LacCer, Gb_3_, and Gb_4_ decreased compared to the WT cells (*dotted boxes*). *D*, the GSL spot quantification of HPTLC plates was done using ImageJ software (https://imagej.net/), the amounts were normalized to the WT values. Error bars represent the standard deviations, n = 3. The significance in difference between the WT and the other two groups (KO and GLTP rescue) was tested using a Student’s *t* test: ∗*p* < 0.05, ∗∗*p* < 0.01. GLTP, glycolipid transfer protein; HPTLC, high-performance thin-layer chromatography.

GLTP was completely absent in all the analyzed GLTP KO cell lines (Fig. 1*A*). In culture, the WT (Cas9 control) lines and the GLTP KO cell lines chosen for glycosphingolipid level analysis (GLTP KO1 and GLTP KO2) showed no apparent morphological differences (Fig. 1*B*). The KO cell lines had reduced levels of GlcCer, LacCer, globosides Gb_3_, and Gb_4_ compared to the WT controls (Fig. 1, *C* and *D*), indicating that the cellular levels of these glycosphingolipids were tied to the expression levels and presence of GLTP in the cells. When analyzing GLTP rescue cells (GLTP KO cells with GLTP production returned through transient plasmid gene transfection) an increase in LacCer, Gb_3,_ and Gb_4_ levels is seen, as compared to GLTP KO cells. This could be due to GlcCer being metabolized to more complex glycosphingolipids (GSLs) such as LacCer, Gb_3,_ and Gb_4_ differently in the two cell types. The amount of GlcCer, on the other hand, remains unchanged if GLTP KO and GLTP rescue cells are compared (Fig. 1*D*). Changes in the ganglioside levels were not detected in this setup (Fig. 1*C*). The membrane phospholipid classes sphingomyelin, phosphatidylcholine, phosphatidylethanolamine, and phosphatidylinositol, as well as cholesterol migrating with the solvent front, did not show any detectable changes in the lipid profiles (Fig. S2). The GSLs levels in the GLTP KO cells where either the GLTP FFAT or the GLTP W96A mutant has been introduced did not show any detectable changes compared to the WT cells (Fig. S2).

### UBE2L6/UBCH8 and carbonic anhydrase 5A were the most upregulated and downregulated genes in GLTP KO HeLa cells

**Figure 2:**
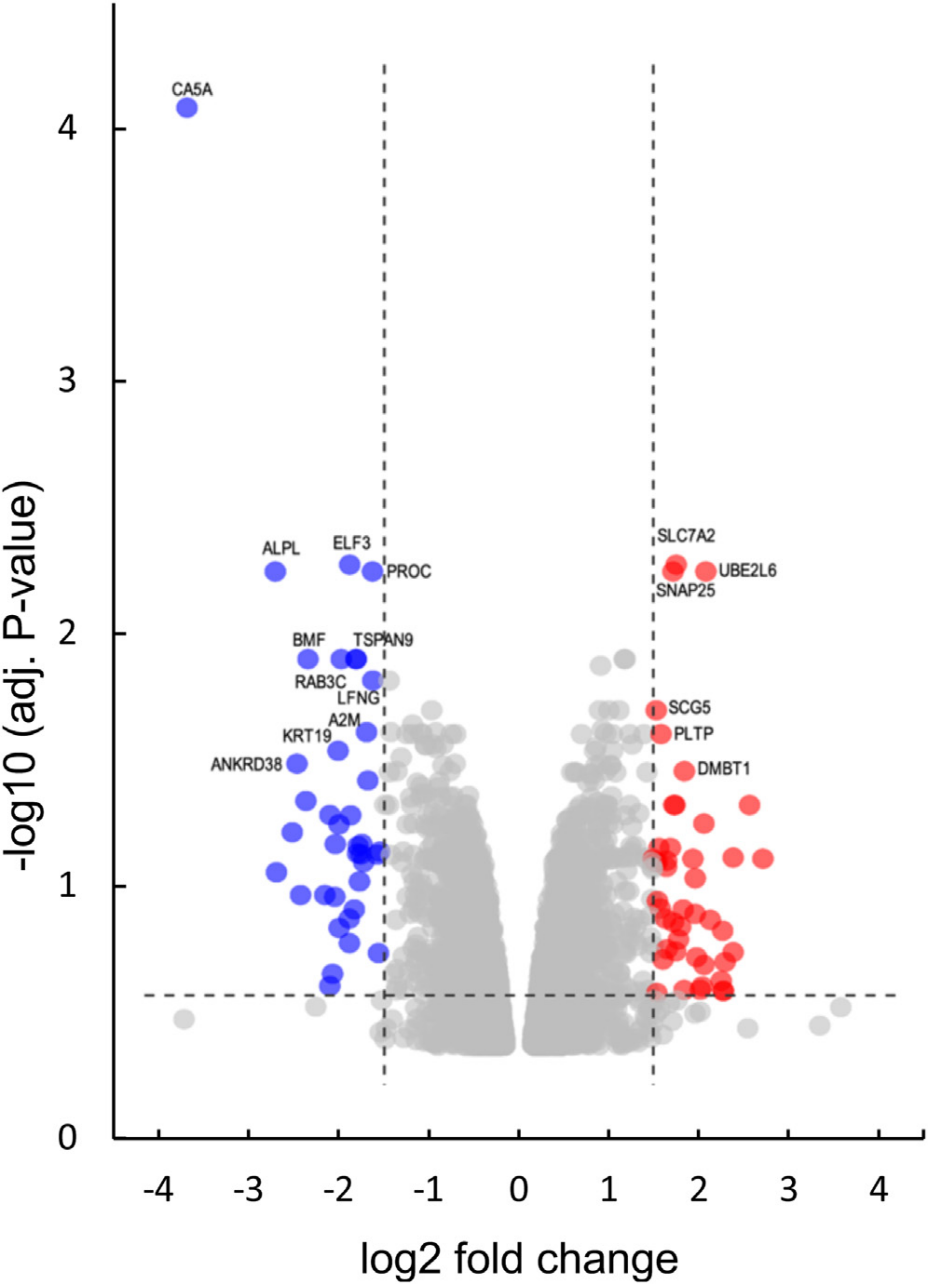
**A volcano plot of genes altered by GLTP KO in HeLa cells.** Genes upregulated with more than 1.5-fold change compared to WT cells with an adjusted *p*-value less than 0.5 are depicted with *red dots* and those downregulated with identical fold change and *p*-value are with *blue dots*. All other genes in the array that were not found to be significantly altered are in *gray dots*. GLTP, glycolipid transfer protein.

To gain a comprehensive understanding of the molecular events involved in the knockout of the GLTP gene in HeLa cells (three clones: GLTP KO1, GLTP KO2, and GLTP KO3), we used the Illumina HumanHT-12 v4 Expression beadchip array to analyze the gene expression profiles. The data presented are the average gene expressions for the three different clones (KO1, KO2, and KO3) *versus* three WT HeLa cell controls (Cas9 GLTP control). The main transcripts that were affected by the GLTP gene knockout are presented in Table 1. The top downregulated gene is carbonic anhydrase 5A—a mitochondrial enzyme involved in ureagenesis and gluconeogenesis. The top upregulated gene is the ubiquitin/ISG15-conjugating enzyme E2 L6 (UBE2L6/UBCH8), catalyzing the ubiquitination of proteins (18). Recent data suggested that UBE2L6 is a consensus core gene in SARS-CoV2 infected cells (19). The overall outcome of the gene expression analysis is presented in a volcano plot with the most significantly downregulated and upregulated genes indicated (Fig. 2).

**Table 1:** The top transcripts affected in GLTP KO1-3 HeLa cells (arranged according to the LogFC value)

Protein code	Definition	LogFC	Adj. *p*
Upregulated genes			
UBE2L6	ubiquitin/ISG15-conjugating enzyme E2 L6 (aka UBCH8)	2.087	0.0053
DMBT1	deleted in malignant brain tumors 1	1.847	0.0361
SLC7A2	solute carrier family 7 member 2	1.756	0.0049
SNAP25	synaptosome associated protein 25	1.721	0.0053
PLTP	phospholipid transfer protein	1.587	0.0253
SCG5	secretogranin V	1.533	0.0201
VEGFC	vascular endothelial growth factor C	1.43	0.0365
SMOX	spermine oxidase transcript variant 4	1.396	0.0253
FHL2	four and a half LIM domains 2	1.265	0.0279
IRF2BP2	interferon regulatory factor 2 binding protein 2	1.237	0.0253
HTRA1	HtrA serine peptidase 1	1.187	0.0122
CXCR4	C-X-C motif chemokine receptor transcript variant 1	1.175	0.0122
EML4	echinoderm microtubule associated protein like 4	1.081	0.0365
CSGALNACT1	chondroitin sulfate N-acetylgalactosaminyltransferase 1	1.048	0.0297
ADARB1	adenosine deaminase, RNA specific B1	1.044	0.0253
MCOLN3	mucolipin 3	1.036	0.046
SMOX	spermine oxidase transcript variant 4	1.03	0.0342
C21ORF63	eva-1 homolog C	1.022	0.0342
FOS	Fos proto-oncogene, AP-1 transcription factor subunit	1.006	0.0201
GLT8D2	glycosyltransferase 8 domain containing 2	0.971	0.0361
RIMKLB	ribosomal modification protein rimK like family member B	0.969	0.025
CXCR4	C-X-C motif chemokine receptor 4 transcript variant 2	0.93	0.0242
IFIH1	interferon induced with helicase C domain.	0.914	0.0339
ADARB1	adenosine deaminase, RNA specific B1	0.912	0.013
C17ORF96	chromosome 17 open reading frame	0.9	0.0201
TMEM194A	nuclear envelope integral membrane protein 1	0.881	0.0486
STAMBPL1	STAM binding protein like 1	0.865	0.0285
ACSL4	acyl-CoA synthetase long-chain family member 4	0.862	0.0342
CAND2	cullin associated and neddylation dissociated 2 (putative)	0.846	0.0297
SEZ6L2	seizure related 6 homolog like 2 transcript variant 2	0.843	0.0396
PKD2	polycystin 2, transient receptor potential cation channel	0.821	0.0374
DTWD1	DTW domain containing 1	0.783	0.037
CHEK2	CHK2 checkpoint homolog (*S. pombe*) transcription variant	0.768	0.0402
TMEM194B	nuclear envelope integral membrane protein 2	0.7	0.0253
TAPBPL	TAP binding protein like	0.643	0.0365
LEPREL2	prolyl 3-hydroxylase 3	0.629	0.0365
FAM92A1	family with sequence similarity 92 member A1	0.59	0.0365
Downregulated genes			
CA5A	carbonic anhydrase 5A	−3.702	0.0001
ALPL	alkaline phosphatase, liver/bone/kidney	−2.717	0.0053
KRT19	keratin 19	−2.475	0.0336
ANKRD38	KN motif and ankyrin repeat domains 4	−2.375	0.0482
BMF	Bcl2 modifying factor	−2.353	0.0122
A2M	alpha-2-macroglobulin	−2.017	0.0297
ELF3	E74 like ETS transcription factor 3	−1.887	0.0049
RAB3C	RAB3C, member RAS oncogene family	−1.819	0.0122
TSPAN9	tetraspanin 9	−1.812	0.0122
LFNG	LFNG O-fucosylpeptide 3-beta-N-acetylglucosaminyltransferase	−1.699	0.0248
MYPN	myopalladin	−1.685	0.0396
PROC	protein C, inactivator of coagulation factors Va and VIIIa	−1.633	0.0053
KRT13	keratin 13	−1.629	0.015
FAM46B	family with sequence similarity 46 member B	−1.496	0.05
ALDH1A2	aldehyde dehydrogenase 1 family member A2	−1.447	0.015
CAMKK1	calcium/calmodulin dependent protein kinase kinase 1	−1.444	0.0365
GAGE12C	G antigen 2E	−1.429	0.0248
EML1	echinoderm microtubule associated protein like 1	−1.359	0.0361
ATP2B4	ATPase plasma membrane Ca2+ transporting 4	−1.313	0.0316
GPER	G protein-coupled estrogen receptor 1	−1.248	0.0253
LMO4	LIM domain only 4	−1.21	0.0467
IGFN1	immunoglobulin-like and fibronectin type III domain containing 1	−1.185	0.023
MYPN	myopalladin	−1.182	0.0253
KLHL4	kelch-like 4 (*Drosophila*) (KLHL4), transcript variant 2.	−1.133	0.0404
NUMBL	NUMB like, endocytic adaptor protein	−1.09	0.0417
GAGE6	G antigen 6 (GAGE6), mRNA.	−1.089	0.0467
CDKN2A	cyclin dependent kinase inhibitor 2A	−1.088	0.045
S100A2	S100 calcium binding protein A2	−1.087	0.0253
ATP2B4	ATPase plasma membrane Ca2+ transporting 4	−1.082	0.025
ATP2B4	ATPase plasma membrane Ca2+ transporting 4	−1.047	0.0279
GSN	Gelsolin	−1.039	0.0282
CACHD1	cache domain containing 1	−1.039	0.045
SSBP3	single stranded DNA binding protein 3	−0.972	0.0201
GAGE12G	G antigen 2E	−0.961	0.0279
RPL29	ribosomal protein L29	−0.96	0.0467
RPS7	ribosomal protein S7	−0.927	0.0248
CGN	cingulin	−0.927	0.0289
CORO6	coronin 6	−0.901	0.0422
SEPW1	selenoprotein W1	−0.887	0.04
FABP5	fatty acid binding protein 5	−0.847	0.0342
PALMD	palmdelphin	−0.845	0.0279
C1ORF50	chromosome 1 open reading frame 50	−0.771	0.0464
TRIM47	tripartite motif containing 47	−0.767	0.0361
ATAD3A	ATPase family, AAA domain containing 3A	−0.753	0.0253
P4HA2	prolyl 4-hydroxylase, alpha polypeptide II, transcript variant 2.	−0.735	0.0461
TMEM93	EMC6, ER membrane protein complex subunit 6	−0.711	0.0336
GSTM2	glutathione S-transferase M2 (muscle), mRNA.	−0.58	0.0499
CCDC34	coiled-coil domain containing 34	−0.561	0.458

The table shows the protein code, definition, base 2 logarithm transformation of fold change (logFC), and adjusted *p*-value.

### GO analysis showed that GLTP KO upregulated cellular components of the endomembrane system and downregulated cellular components in the cytosol

**Figure 3:**
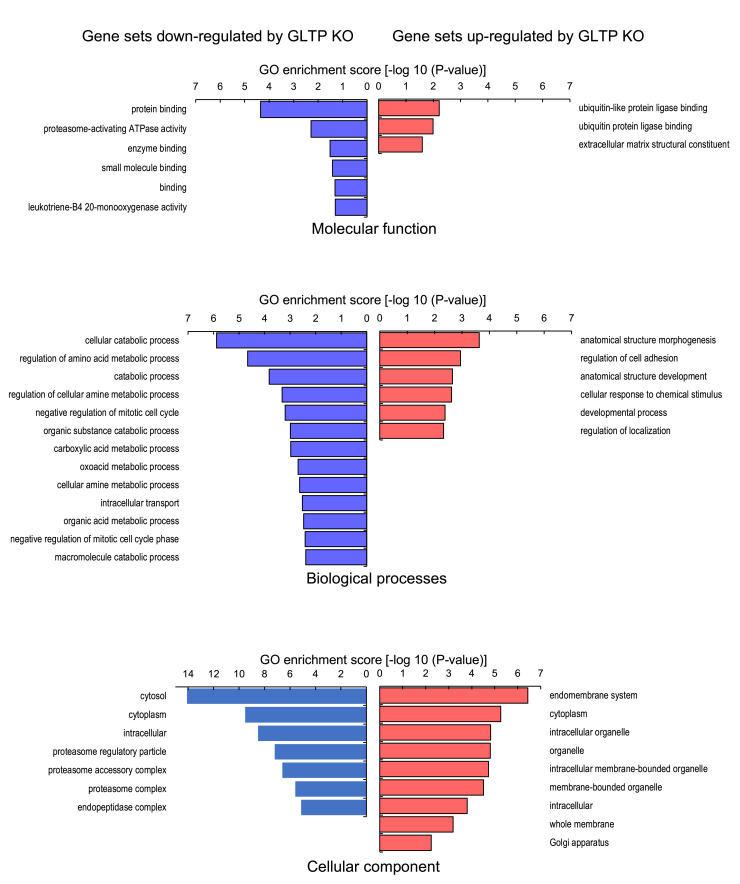
**Downregulated and upregulated gene sets in GLTP KO cells.** Gene set enrichment analysis showing downregulated processes (*blue*) and upregulated processes (*red*) for GLTP KO HeLa cells with the most significant set of genes compared to GLTP WT cells. Of the analyzed genes, the most significant (adjusted *p*-value < 0.2) genes were picked from among the most upregulated and downregulated ones and subjected to GO-term identification. GLTP, glycolipid transfer protein; GO, Gene ontology.

The most significant upregulated and downregulated genes were selected and subjected to GO-term identification analysis. Figure 3 shows the downregulated and upregulated processes for the KO cells, respectively, as identified by the gene set enrichment analysis. Gene sets that were downregulated in the KO cells were, according to the GO enrichment analysis, associated with molecular functions such as proteinbinding and proteasome-activating ATPase activity (proteasomal protein catabolic process), biological processes such as cellular catabolic processes and regulation of cellular amino acid metabolic processes, and gene sets associated with intracellular cellular components and the cytosol. Meanwhile, according to the GO analysis, gene sets that were upregulated in KO cells were associated with the biological processes of anatomical structure generation and regulation of cell adhesion; gene sets associated with molecular functioning, such as ubiquitin (and ubiquitin-like) protein ligase binding; and gene sets associated with cellular components such as the endomembrane system, cytoplasm, and organelles.

### Knockout of GLTP increased cell migration and motility rate

**Figure 4:**
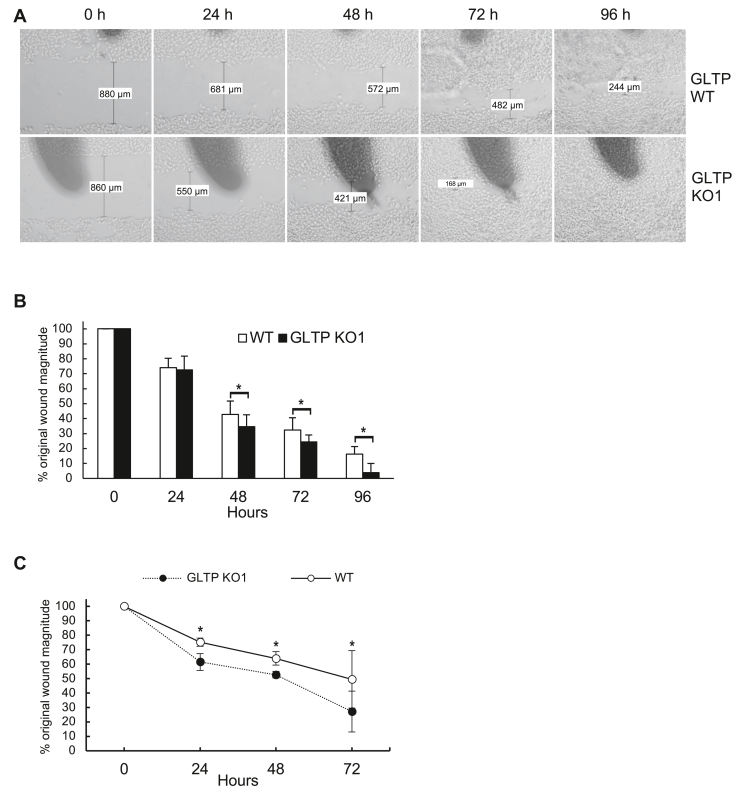
**GLTP KO cells display increased motility in wound-healing assays.** A wound-healing assay was performed on WT and GLTP KO1 HeLa cells. *A*, the experiments were repeated six times with similar results and representative micrographs (10×) are shown. *B*, wound closure was photographed and quantified and the remaining gap between the migrating cells, from the opposing wound edges, is expressed as a percentage of the initial magnitude of the scratch. *C*, wound closure for mitomycin (10 μg/ml) pretreated HeLa cells. The significance difference between the two groups was tested using a Student’s *t* test: ∗*p* < 0.05, ∗∗*p* < 0.01. GLTP, glycolipid transfer protein.

The GO analysis of the transcriptomes of KO cells indicated that regulation of cell adhesion could be upregulated by GLTP knockout (Fig. 3); therefore, the effect of GLTP knockout on the migration of HeLa cells was investigated using a wound-healing assay. Confluent monolayers of WT1 and KO1 cells were scratched and the wound closure (approx. 800 μm) was monitored over time (Fig. 4). A mean rate of wound closure was calculated based on the images taken at 24-h intervals and was determined as a rate of 9.0 μm ± 1.3 μm/h for WT HeLa cells and 9.7 μm ± 1.8 μm/h for GLTP KO HeLa cells. The growth and migration rates of the KO cells were thus determined, on average, to be approximately 8% faster than those of the WT cells. To reduce the risk of cell proliferation confounding the migration studies, we also used mitomycin C to inhibit DNA synthesis and consequently cell proliferation (20). In Figure 4*C* we show that the wound closure was still significantly faster for the GLTP KO cells compared to the WT. This confirms that cell division was likely not the cause of the wound closure but rather cell motility and migration.

### Knockout of GLTP did not impact cell growth rates

**Figure 5:**
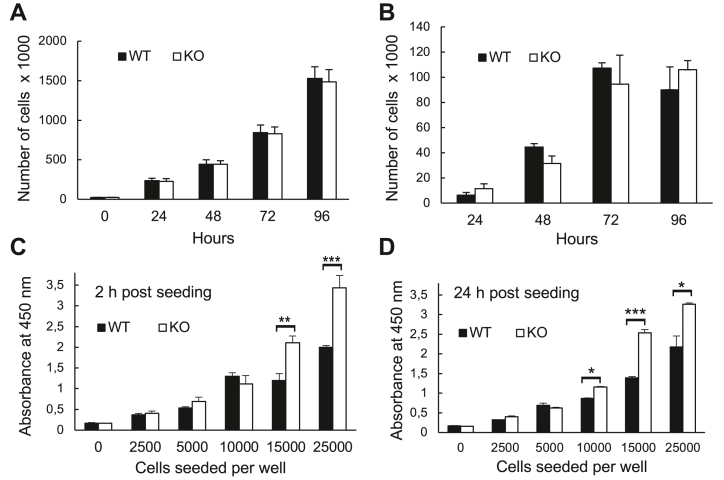
**GLTP KO does not impact the cell growth rates but affects cell metabolism.** Cell growth rates of WT and GLTP KO1 HeLa cells. *A*, manual cell counts to determine the amount of WT1 and GLTP KO1 HeLa cells at different time points. *B*, cell counts determined with the CyQuant assay for WT and GLTP KO1 HeLa cells. *C* and *D*, amounts of living cells measured with a Dojindo Cell Counting Kit-8 (CCK8) at 2 h (*C*) and 24 h (*D*) after seeding, indicated by the absorbance of the formazan dye at 450 nm. (n ≥ 3 independent experiments; means ± SEMs). The significance was tested using a Student’s *t* test: ∗*p* < 0.05, ∗∗*p* < 0.01, ∗∗∗*p* < 0.005. GLTP, glycolipid transfer protein.

The cell growth of WT and KO HeLa cells was also analyzed and counted at 24-h intervals and the extents of growth are presented in Figure 5*A*. While a very slight decrease seemed to occur in the proliferation rate of KO HeLa compared to WT HeLa cells, the difference was so minor that GLTP knockout could not be established as affecting cell proliferation to any significant degree. Similarly, the CyQUANT cell growth assay, which measures total cellular DNA amount, did not reveal any significant differences in proliferation rates between WT and KO cells (Fig. 5*B*). The CCK8 assay, however, provided unexpected results. Even though the same amounts of cells had been seeded to the wells, the first absorbance measurement after cell seeding repeatedly showed a greater absorbance in the wells containing KO cells than the ones containing WT cells. This indicates a larger number of KO cells but because this occurred no more than 2 h after the cells were initially seeded at identical concentrations (Fig. 5*C*), this was highly unlikely. Manual cell counting showed no noticeable discrepancies in the cell numbers. This discrepancy persisted 24 h later, with manual counting of the cells once again confirming that the KO cells had not proliferated to a degree that would explain this discrepancy through the number of viable cells alone (Fig. 5*D*). However, an explanation may be that, unlike the CyQUANT assay, CCK8 does not measure an absolute amount of any cellular component; instead, it measures the number of viable cells by using cellular metabolism to convert a precursor salt into a light-absorbing dye. The reduction of the WST-8 (water-soluble tetrazolium salt, 5-(2,4-disulfophenyl)-3-(2-methoxy-4-nitrophenyl)-2-(4-nitrophenyl)-2H-tetrazolium monosodium salt) precursor tetrazolium salt into the formazan dye is mediated *via* the oxidization of NAD(P)H to NAD(P)^+^ in cells and the conversion rate of salt to dye thus relies on the activity rate of dehydrogenases in cells rereducing NAD(P)^+^ to NAD(P)H. Therefore, a difference in metabolic rate—more specifically, dehydrogenase activity—between the WT and KO cells could, in fact, have caused the WST-8 salt to be converted into formazan dye at a faster rate by KO cells than by WT cells, resulting in the observed discrepancy. The implications of this observed effect led us to study and compare the cellular metabolism of WT and KO cells to better understand and determine the effect of GLTP knockout on cellular respiration and mitochondrial activity.

### Cellular metabolism was altered in GLTP KO cells

**Figure 6:**
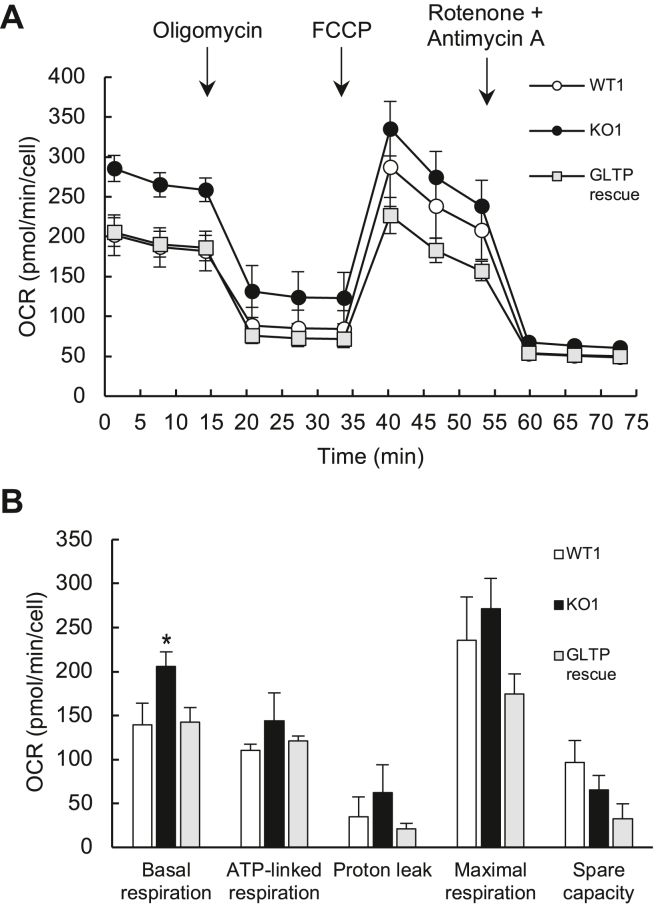
**Oxygen consumption rate (OCR) is reduced in GLTP KO cells.***A*, oxygen consumption rate graphs for WT, GLTP KO, GLTP overexpressing, and GLTP rescue cells; the times of addition of oligomycin, FCCP, and rotenone + antimycin A are indicated with *arrows*. *B*, extracted values for the basal mitochondrial OCR, proton leak, ATP-linked respiration, maximal respiration, and nonmitochondrial respiration. The OCRs were measured in real time using a Seahorse metabolic flux analyzer, as described in the Experimental procedures section. The values represent means ± SDs (n = 15–20). Significance was tested using a Student’s *t* test: ∗*p* < 0.05. GLTP, glycolipid transfer protein.

To measure whether mitochondrial functioning of the KO cells differed from that of the control HeLa cells, we performed a mitochondrial stress assay using a Seahorse metabolic flux analyzer. The results showed that the basal OCR significantly increased in the GLTP KO cells compared to the control HeLa cells (Fig. 6). This explains the discrepancy observed in the absorbance values in the CCK8 signal of equal amounts of WT and GLTP KO cells (Fig. 5, *C* and *D*), corroborating our hypothesis that a difference in metabolic rates is responsible for the difference in the basal OCR levels. Both the KO and HeLa control cells responded and showed a decreased OCR due to the addition of the ATP-synthase inhibitor oligomycin. The mechanisms that couple oxygen consumption and ATP production were similarly affected in both the control and GLTP KO HeLa cells. The difference between the OCR consumption after rotenone and antimycin A addition (nonmitochondrial OCR) and the OCR after oligomycin addition for each cell line indicated the amount of proton leak (21). GLTP KO cells displayed a slight increase in proton leak compared to the other cell lines but the difference was not significant. The addition of the uncoupling agent FCCP (carbonyl cyanide-*p*-trifluoromethoxyphenylhydrazone) caused the proton gradient to collapse and disrupted the mitochondrial membrane potential, leading to an uninhibited electron flow through the electron transport chain and maximum oxygen consumption by complex IV of the electron transport chain (21). This was observed as a rapid OCR increase in both WT and KO cells. The oxygen consumption increase was similar for all observed cell lines, except GLTP rescue cells, where the overall rate decreased (although, again, not significantly compared to WT). Calculating the difference in OCR between basal and maximal respiration reveals the spare capacity of cells (21). Finally, we measured nonmitochondrial respiration by adding rotenone and antimycin A to inhibit the respiratory chain. No significant difference was observed between WT and KO cell lines in this case (Fig. 6).

### GLTP KO and the ΔFFAT-GLTP mutant disrupted the spheroid formation marginally

**Figure 7:**
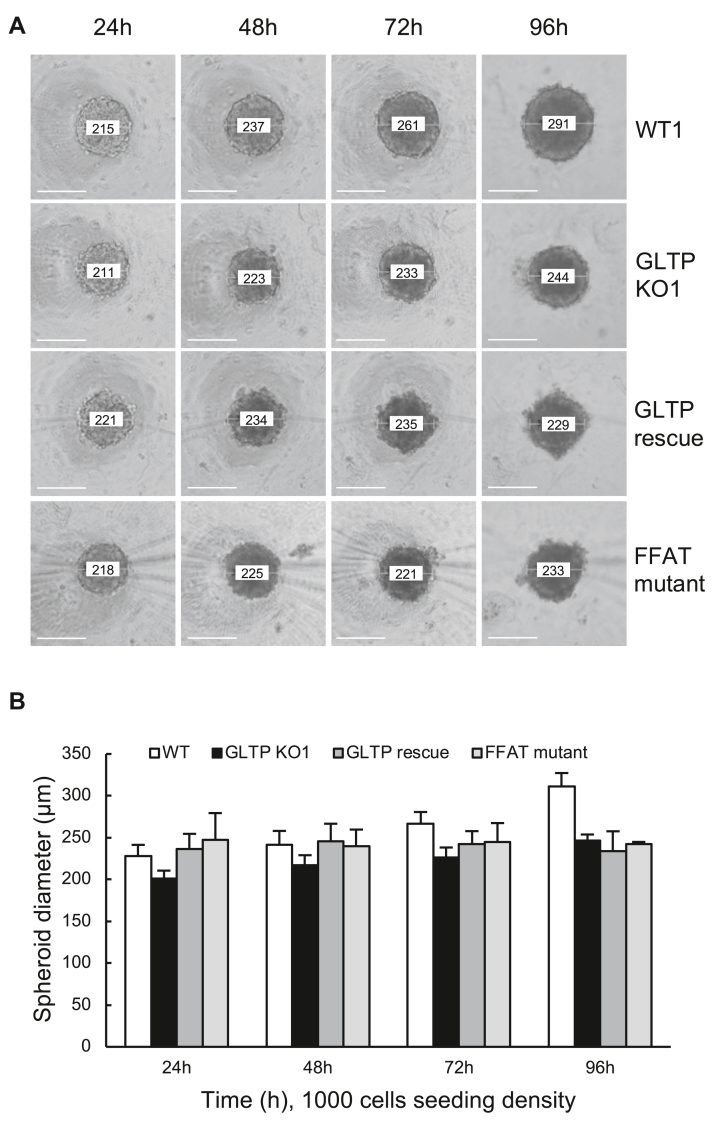
**Effect of GLTP knockout on the growth of HeLa spheroid structures.** Multicellular spheroids of different HeLa cell lines established from 1000 cells seeded per well in Corning round-bottomed 96-well ultra-low-attachment plates, shown at different time points. WT1, GLTP KO1, WT GLTP rescue, and FFAT mutant GLTP expressing cells were used to grow spheroids. *A*, representative images of cellular morphological changes during 96 h of culture observed under a contrast microscope. The scale bars represent 100 μm. *B*, spheroid diameter of cells at different time points postseeding of 1000 cells/well. Error bars represent the standard deviations, n = 3 to 6. GLTP, glycolipid transfer protein.

To examine the growth properties of the GLTP KO cells, we used a spheroid growth assay. The growth of spheroids for WT HeLa, KO1, GLTP rescue, and ΔFFAT-GLTP mutant-expressing HeLa cells was observed at 24-h intervals over a 96-h period, as shown in Figure 7. The morphological differences between the WT and KO spheroid structures were minor, with GLTP KO cells growing into slightly smaller spheroids. The structures formed by WT HeLa cells were smooth and spherical, while the KO cell spheroids were generally more unevenly shaped. In both cases, however, the spheroid structures were cohesive and mostly spherical (Fig. 7). As described earlier, the ΔFFAT-GLTP mutant possesses a mutation in its FFAT domain which renders it incapable of interacting with VAP-A on the ER surface (9, 22). Since this interaction was predicted to affect vesicular transport in cells (12, 13), inhibiting it could also have noticeable effects on the spheroidal growth of the cells. However, the effect on spheroid formation of transiently expressing the ΔFFAT-GLTP mutant was minor. The resulting ΔFFAT-GLTP mutant expressing spheroids were more unevenly shaped and even smaller in size than their counterparts consisting of WT cells or GLTP KO cells but the structures were still cohesive and spheroidal (as shown in Fig. 7).

However, in the case of GLTP-rescue cells, transiently transfecting KO cells with WT GLTP did not result in the spheroids mimicking the morphology of WT spheroids. Instead, GLTP rescue resulted in the apparent failure of the cells to form structurally cohesive spheroids, as shown in Figure 7. Mock transfections carried out by transfecting KO cells with a blank plasmid (pcDNA3.1+, 10 μg of plasmid per 100-mm cell plate, preseeding) did not display a similar effect, ruling out an effect of electroporation on the cells as the cause (data not shown).

### Knockout of GLTP disrupted vesicle trafficking

**Figure 8:**
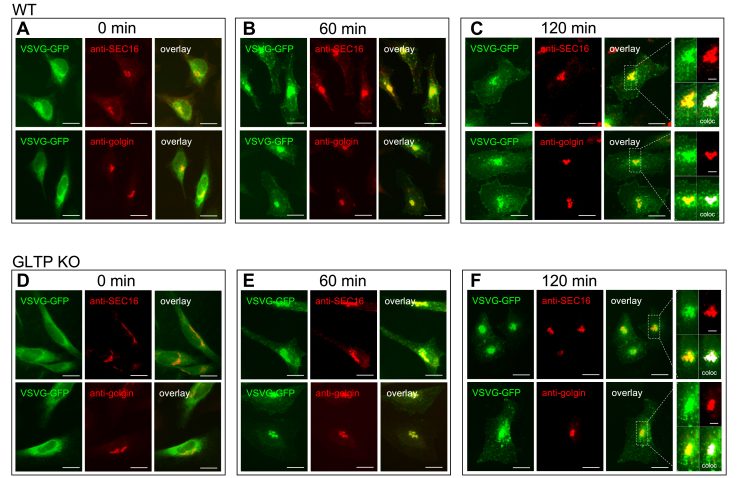
**GLTP KO disrupts vesicle trafficking.** Representative fluorescence images of VSVG-GFP fusion protein distribution in HeLa cells at 60-min intervals from a temperature shift from nonpermissive temperature (40 °C) to permissive temperature (32 °C). VSVG-GFP in *green* and anti-SEC16 and anti-golgin in *red*. (*A*), (*B*), and (*C*) are WT HeLa cells and (*D*), (*E*), and (*F*) are GLTP KO HeLa cells.The scale bars represent 100 μm. In (*C*) and (*F*) the subpanels on the *right* are higher magnifications (2×) of the areas outlined in *white* (The scale bars represent 50 μm). The overlay panels show merged *green* and *red images*. The coloc panel displays a colocalization mask on which pixels where *green* and the *red channels* colocalize are shown in *white*. GLTP, glycolipid transfer protein; VSVG, vesicular stomatitis virus protein G.

The effect of GLTP knockout on vesicle trafficking was studied using vesicular stomatitis virus protein G (VSVG)-GFP-expressing cells following the temperature shift to the permissive temperature of the construct (23, 24, 25). To validate the function of the assay, expression and transport of the VSVG-GFP construct was compared in WT cells and GLTP KO cells, with additional organelle staining against the endoplasmic reticulum exit sites (ERES) and against the Golgi being carried out as well. In both the WT HeLa and GLTP KO HeLa cells the fluorescent VSVG-GFP construct was retained in the ER and at the ERES at the initial time point 0 min (Fig. 8, *A* and *D*). At 60 min after the temperature shift from nonpermissive to permissive temperature (40–32 °C) (23, 24, 25), the fluorescent VSVG-GFP construct was observed to accumulate strongly at both nucleus-adjacent and more distal ERES in WT cells, as well as beginning to accumulate at the plasma membrane, indicating normally functioning vesicle-mediated transport of the construct (Fig. 8, *B* and *E*). After 120 min, the VSVG-GFP construct was observed to have accumulated even more strongly at the entire extent of the plasma membrane, as expected. However, in the GLTP KO HeLa cells the VSVG-GFP construct was retained at the ERES at a much higher extent than in the WT cells throughout the observed time, with much less of the construct reaching the plasma membrane. This, in turn, indicated that the absence of GLTP disrupted the vesicular trafficking pathway preventing the vesicle-mediated transport of the VSVG-GFP construct from the ER to the plasma membrane. The change in the fluorescence intensity, and hence the transport from the ERES toward the plasma membrane, is shown in Figs. S3 and S4 using 3D surface plot analysis. Based on the location of the intensity of the VSVG-GFP we determined that most (>90%) of the VSVG-GFP remained in the ERES in the GLTP KO cells (Fig. S3*D*), whereas in the WT GLTP cells less than 50% of the VSVG-GFP fluorescence intensity remain in the ERES after 120 min (Fig. S3*B*). The estimation is based on analysis of several cell images. In addition, through using the anti-SEC16 and anti-golgin-97 antibodies (both shown in red in Fig. 8) we showed that the VSVG-GFP construct localized significantly more with the SEC16 ERES marker compared to the Golgi marker golgin-97 (Fig. 8, *C* and *F*), confirming that the construct is indeed retained primarily at the ERES and not in the Golgi apparatus.

**Figure 9:**
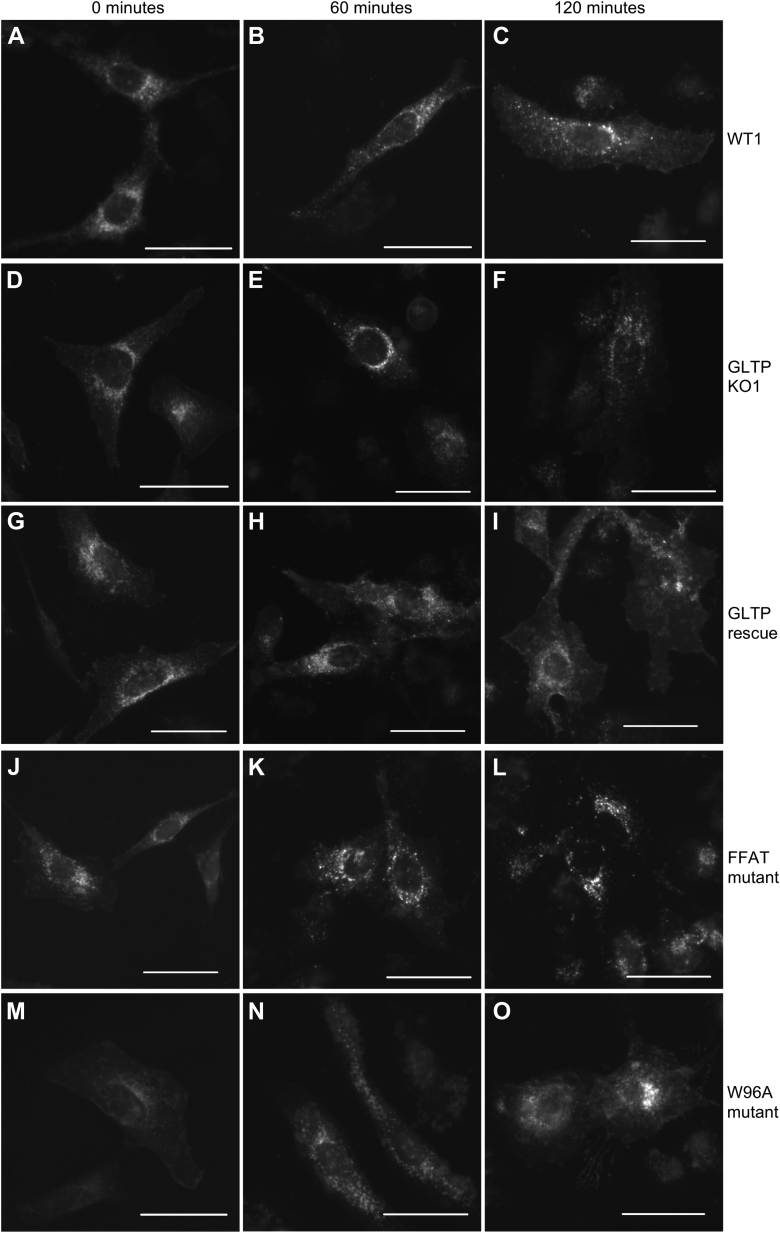
**The GLTP FFAT mutant cannot sustain VSVG-GFP trafficking.** Representative fluorescence images of VSVG-GFP fusion protein distribution in HeLa cells at 60-min intervals from a temperature shift from nonpermissive temperature (40 °C) to permissive temperature (32 °C). The VSVG-GFP construct was retained at ER exit sites and was released and sorted through the cell toward the plasma membrane. *A*–*C*, WT HeLa cells. *D*–*F*, GLTP KO HeLa cells. *G*–*I*, GLTP rescue HeLa cells. *J*–*L*, GLTP FFAT-domain mutant knock-in HeLa cells. *M*–*O*, GLTP W96A point mutant knock-in HeLa cells. The scale bars represent 200 μm. ER, endoplasmic reticulum; GLTP, glycolipid transfer protein; VSVG, vesicular stomatitis virus protein G.

Next, we studied the effect of GLTP mutants on VSVG-GFP trafficking. A similar effect as in WT cells (Fig. 9, *A*–*C*) was observed in the GLTP-rescue cells, with the construct accumulating first at the ERES and thereafter increasingly at the plasma membrane, indicating that the transient transfection of WT GLTP into KO cells was successful in restoring GLTP expression and functionality to resemble that of WT cells (Fig. 9, *G*–*I*). In the KO cells, as seen previously (Fig. 8, *D*–*F*), no such effect was observed even at 120 min after the temperature shift, with the VSVG-GFP construct remaining strongly accumulated at the ERES throughout the observed time (Fig. 9, *D*–*F*). However, most interestingly, the KO cells in which ΔFFAT-GLTP was transiently expressed showed a practically identical effect (Fig. 9, *J*–*L*) to that seen in the KO cells. It thus appeared that the GLTP–VAP interaction, absent in ΔFFAT-GLTP mutants, was directly implicated as a cause for the observed effects on vesicle transport in the cells, in line with previous predictions (6). The effect seen in KO cells transiently expressing W96A-GLTP (Fig. 9, *M*–*O*) was less easy to interpret, because while some amount of the VSVG-GFP construct appeared to have been transported to the plasma membrane, a significant amount also remained at the ERES at the 120-min time point. Since W96A-GLTP can interact with VAPs, unlike ΔFFAT-GLTP, the transport of VSVG-GFP to the plasma membrane in this case supported the interpretation of GLTP–VAP interactions as important for the proper functioning of the vesicle transport system. However, the inability of W96A-GLTP to bind glycolipids, an interaction shown to affect interactions with VAP (6), quite possibly led to other disruptions in its function, leading disturbance in the vesicle trafficking.

## Discussion

The aim of this study was to characterize the effects of GLTP gene knockout in cells and thereby gain insight into the *in vivo* role of this protein. The genes most affected by GLTP knockout were identified and selected *via* gene ontology term analysis. For instance, cell motility was predicted to be upregulated in the GLTP KO cells and wound-healing assays confirmed this, with KO cells closing a gap in a confluent monolayer approximately 8% faster than GLTP WT cells. Although GLTP KO cells grew at almost the same rate as WT cells, we observed a much larger difference in cellular metabolism between WT and KO cells. Basal mitochondrial respiration markedly increased when the GLTP gene was knocked out—an effect that could be reversed by reintroducing GLTP. ATP-linked respiration was not affected, indicating that the differences observed in the basal respiration were not ATP-linked. The slight increase in proton leak in the KO cells could have been due to disrupted mitochondrial ATP production, although the changes were not significant. GLTP’s observed interaction with VAP-A and its apparent role in vesicular trafficking (6, 26) could have caused a reorganization of substrates/fuel available for the electron transport chain due to a decrease in GLTP-mediated vesicular trafficking from the ER, leading to the observed mitochondrial respiration effects.

The GLTP KO cells showed alterations in the lipidome. The levels of GSLs decreased in response to GLTP knockout, supporting our previous hypothesis that GLTP has a regulatory effect on the GSL levels in cells, as well as acting as a transport protein for the same (6, 27). The exact mechanism by which GLTP regulates the levels of GSLs in the cell is still unclear but it is likely that GLTP does so *via* interactions with other proteins and protein complexes at the ER/Golgi interface (26). One potential interaction partner for this is VAP at the ER surface. It has been demonstrated that the interaction of GLTP with VAP is inhibited when a GSL substrate (*e.g.*, GlcCer) is bound to GLTP (6). Increased levels of GSL would lead to increased GLTP-GSL binding and diminished GLTP–VAP complex formation and vice versa, with GLTP–VAP complexes forming with greater frequency and for longer periods of time when less GSL is present for GLTP to bind. Speculatively, this interaction could be (even if indirectly) tied to the maintaining of GSL homeostasis in the cell. Another speculative explanation could be that the presence of GLTP at the ER *via* VAP interaction places it near the ceramide synthesis pathway in the ER, allowing it to begin affecting GSL synthesis already at the precursor stage.

However, GLTP–VAP complex formation is unlikely to alone be a directly influencing factor on GSL synthesis, as GLTP is only one among a plethora of interacting partners of VAP. VAP-A and its homolog VAP-B recognize and interact with proteins containing FFAT or FFAT-like motifs, with their interacting partners numbering at an estimated 100 cytosolic protein domains (22, 28, 29). VAP-A and VAP-B are the only known ER-proteins that anchor other proteins on the ER surface. Through these interactions, very different protein complex organizations and functions are formed; some complexes are retained at the ER, whereas others bridge the ER with various organelle membranes, forming membrane contact sites (MCS). The exact role of GLTP in MCSs is as of yet uncertain. However, through its interaction with VAP (itself a key component in MCS complexes), GLTP is most likely also an interacting part in MCS protein complexes.

It remains unclear why the expression of WT GLTP in GLTP KO cells does not result in spheroids formed from these cells mimicking the morphology of WT cell spheroids but instead in apparently noncohesive, irregularly shaped spheroids. The same occurs in spheroids composed of KO cells expressing ΔFFAT-GLTP, although these spheroids appear more regularly shaped and cohesive than WT GLTP rescue cell spheroids. The implications of these results are still unclear and fall beyond the scope of this paper, warranting further study.

With ΔFFAT-GLTP sharing all functions of WT GLTP except for its ability to interact with VAP, this specific interaction is implicated when ΔFFAT-GLTP expression achieves a similar apparent disruption of the vesicular transport as the complete absence of GLTP does. It can of course be debated whether the apparent disruption of vesicle transport is a true disruption of trafficking altogether, a cargo incorporation issue at the ERES, or indicative of something else. It can also be debated whether it is directly caused by the absence of GLTP–VAP interaction, or if it is a causal effect occurring downstream of this interaction. Regardless of whether the effect is direct or indirect, it appears that the absence of GLTP, as well as the absence of the GLTP–VAP interaction, causes a much higher degree of retention of vesicular cargo at the ERES than is seen in WT cells, by inference implying a disruption of vesicle traffic originating from the ER. This also effectively indicates that a downregulation of cellular sphingolipid levels (*i.e.*, as induced by the knockout of GLTP) is not the cause of VSVG transport disruption, as has been suggested previously by the Pagano group (30) as ΔFFAT-GLTP expression disrupts VSVG trafficking while being otherwise functionally identical to WT GLTP, including in its effect on cellular GSL levels (Fig. S2).

The expression of W96A-GLTP does not disrupt vesicle trafficking in the same way as GLTP knockout or ΔFFAT-GLTP expression. W96A-GLTP is fully capable of interacting with VAP, the interaction which appears to drive functional cargo loading and/or vesicle transport altogether is intact. We observe that while protein cargo is trafficked out of the ER in vesicles as normal in W96A-GLTP-expressing cells, a fraction of cargo which is significantly higher than in WT and GLTP rescue cells is also retained in the ER and at the ERES. More than likely, the effects seen caused by the expression of W96A-GLTP are distant effects, caused by decoupling GLTP from its substrate-binding-associated regulation mechanism and making its protein–protein interactions essentially random, at the expense of other, controlled interactions at the ER-Golgi contact sites.

In conclusion, we have for the first time demonstrated *in vivo*, that GLTP is essential for vesicle trafficking. GLTP is shown to affect many aspects of the biology of the cell, from regulating its glycosphingolipid homeostasis to affecting metabolism and vesicular trafficking. However, the interactome of GLTP is still very much unclear (26, 31), with its confirmed interactions extending to its interaction with VAP, as well as its GSL-binding function. The GLTP interaction with VAP also makes GLTP a candidate protein in the protein complexes of the MCS, in which VAP is a key participant. Studying the possible roles of GLTP in the MCS complexes may elucidate the effects that GLTP appears to have on the cell.

## Experimental procedures

### Cell culture

HeLa cells (ATCC CCL-2; LGC Standards) were cultured at 37 °C in a 5% CO_2_ atmosphere in Dulbecco’s Modified Eagle’s medium (DMEM) supplemented with penicillin (50 U/ml), streptomycin (50 U/ml), 4 mM L-glutamine, and 10% fetal calf serum.

### Guide RNA constructs

The GLTP protein was stably knocked out in HeLa cells by double RNA-guided clustered regularly interspaced short palindromic repeats (CRISPR) with Cas9-nickase endonuclease activity. Guide RNAs (gRNA), intended to target the endogenous GLTP gene, were designed with the CRISPR Ensembl design tool (http://www.ensembl.org) and the designed gRNA primers are listed in Fig. S1.

### Generation of stable GLTP KO cell lines

HeLa cells were seeded to 12-well plates and transfected with Lipofectamine LTX. Transfection mixtures contained 0.2 μg of gRNA Guide A and 0.2 μg of rRNA Guide B, combined with 0.6 μg of Cas9n. Cas9 control GLTP cells were prepared without adding the GLTP-specific gRNA constructs to the transfection mixture. At 6 h after transfection, the cells were trypsinized and moved to 6-well plates (1:5 split). Single-cell clones were cultured in 96-well plates for 2 to 3 weeks to generate stable KO cell lines. The effectiveness of the knockout was analyzed using Western blot analysis with an anti-GLTP antibody, as described earlier in other studies (6, 32). The mutations generated were identified in GLTP complementary DNA (cDNA) by extracting the genomic DNA using the NucleoSpin Tissue kit from Macherey-Nagel and then amplifying the exon 1 region using PCR. Q5 High-Fidelity DNA Polymerase (New England BioLabs; M0491S) and the primers (Forward, TAAGCAAAGCTTATGAACCGGCGCACCTCCTCT; Reverse, ACGTCGGGATCCAGAGATGGTTAGGGGATGCTC) were used to generate the exon 1 region PCR product. The resulting PCR product was inserted into a pcDNA3.1 plasmid between the HindIII and BamHI restriction sites and transformed into DH5α cells for cloning.

### Gene vector transfection for generation of GLTP rescue and mutant protein expressing cells

In order to be able to study the recovery of GLTP function in GLTP KO cells, as well as to be able to overexpress GLTP or express mutant forms of the GLTP protein, plasmid DNA encoding for the relevant genes was transiently transfected into HeLa cells through electroporation with a Neon transfection system (Thermo Fisher Scientific). Cells were grown to confluency on 100-mm culture dishes (to a minimum of 5 × 10^6^ cells), following which the cells were harvested by trypsinization and pelleted. The pellets were then resuspended in resuspension buffer (Thermo Fisher Scientific, proprietary buffer for Neon transfection kit), mixed with plasmid DNA solution (10 μg DNA per plasmid per 5 × 10^6^ cells), and electroporated at Thermo Fisher’s recommended system settings for the relevant cell line (HeLa). The transfected cells were resuspended in DMEM and plated onto new 100-mm culture dishes; for lipid extraction or harvesting for proteins, cells were allowed to recover for 24 h before being harvested. Vectors used were pcDNA3.1, GLTP mutants, ΔFFAT and W96A, full length human GLTP for GLTP rescue assays transfected in GLTP KO cells; and the pEGFP3-VSVG vector for the VSVG-GFP protein construct used in the vesicle transport assays.

### Illumina beadchips array analysis

The total HeLa cell transcriptome analysis was carried out using Illumina HumanHT-12 v4 Expression BeadChip array (Illumina). RNA was isolated from cultures of WT (Cas9 GLTP control) and three different GLTP gene KO clones (termed GLTP KO1, GLTP KO2, and GLTP KO3). 100 ng of total RNA was primed using oligo-dT(T7) primers and converted to cDNA with Invitrogen SuperScript III Reverse Transcriptase. The double-stranded cDNA template was amplified and labeled with *in vitro* transcription to generate multiple copies of biotinylated cRNA. Purified biotinylated cRNA was hybridized to HumanHT-12 v4 expression beadchips for 18 h at 58 °C using a rocking platform at speed 5. Beadchips were washed, blocked, stained with streptavidin-Cy3, and scanned with an Illumina iScan scanner, using the manufacturer’s recommended protocols. The raw data were extracted using Illumina’s GenomeStudio software (https://www.illumina.com).

### GO analysis

Enrichment of genes with a particular annotation was carried out using the g:Profiler web-based toolkit (biit.cs.ut.ee/gprofiler/gost). The functional enrichment analysis was also performed using g:Profiler (version e101_eg48_p14_baf17f0) and a g:set counts and sizes multiple testing correction method with a significance threshold of 0.05 (33). The *p*-value was the probability of obtaining the same annotation cluster with a random selection of genes. The lower the *p*-value, the more likely that the gene enrichment analysis was specific to the gene list and not a random event.

### Lipid analysis

Cellular total lipids were extracted from the tissue culture dishes using hexane-isopropanol (3:2 by volume) as described (34). WT, GLTP KO, and GLTP rescue cells (extracted 24 h after hGLTP transfection) were used for extraction. Lipids were identified using lipid standards, run in parallel with the samples. The high-performance thin-layer chromatography plates with glycosphingolipids were visualized using orcinol spray (0.2% orcinol in a 20% H_2_SO_4_ solution) and developed at 120 °C for 5 min. For other lipids, subsequent iodine or cupric acid staining was used for visualization (34).

### Cell migration assay

Wound-healing assays with WT, GLTP, and KO1 HeLa cells were carried out in 100-mm cell plates, where cells could grow to full confluency before the cell monolayer was scratched with a 1000 μl pipette tip. HeLa cells were also treated with mitomycin C (10 μg/ml) for 2 h to prevent cell proliferation followed by scratch wounding. The resulting scratch was then immediately photographed with an inverted microscope (Leica DMi1) and monitored for at least 72 h while maintaining the culture conditions, with further images taken at 24-h intervals and analyzed with Leica LAS EZ software (https://www.leica-microsystems.com/).

### Cell proliferation assay

To determine whether GLTP gene KO influenced the growth and proliferation rates of HeLa cells, two commercially available kits were chosen to determine cell proliferation. A Dojindo Laboratories Cell Counting Kit-8 (CCK8) and an Invitrogen CyQUANT cell proliferation assay kit (Thermo Fisher Scientific). To supplement these kits, manual counting of cells from microscopy images was chosen as a third approach to study and characterize cell proliferation. For manual counting, the WT and GLTP KO1 cells were seeded to a known concentration (25,000 cells/ml, 250,000 cells total) on 100-mm cell plates and thereafter counted using trypan blue exclusion at 24-h intervals.

The CCK8 assay measures the number of living cells using cellular metabolism to convert a precursor molecule into a dye in the growth medium, the amount of which can be quantified *via* absorbance measurement of the medium. The CyQUANT kit, by contrast, measures the amount of DNA in harvested, lysed cells using a DNA-binding fluorescent dye. The CCK8 assay was carried out according to the recommended kit protocol with no modifications, with a 2-h incubation period after application of the tetrazolium dye precursor (WST-8 precursor) before measurement of light absorbance at 450 nm. Wells were seeded with 25,000 cells each (a concentration of 250,000 cells/ml) and absorbance measurements were taken at 0, 24, and 48 h after seeding (accounting for the incubation time after dye precursor addition).

The CyQUANT assay was likewise carried out according to the recommended protocol. A standard curve was prepared for harvested WT and KO1 cells to calculate the number of cells from the measured fluorescence emission.

### Cellular metabolism assay

To analyze possible differences in cellular metabolism caused by the gene KO, WT, and KO1 cells were subjected to a mitochondrial stress test assay, carried out with a Seahorse XF96 Flux Analyzer (Agilent Technologies). The basal OCR of the cells was recorded, followed by the addition of oligomycin, which inhibits ATP production and yields a proton leak signal. Addition of the mitochondrial uncoupling agent FCCP allows for an unhindered flow of electrons through the electron transfer chain, so that the maximal oxygen consumption of complex IV can be recorded. Finally, to inhibit the respiratory chain and record the nonmitochondrial respiration, rotenone, and antimycin A were added according to the manufacturer’s protocol. Based on these measurements, the basal and maximal OCRs were calculated. The basal respiration, as well the respiration after the addition of oligomycin, FCCP, rotenone, and antimycin were all measured at three consecutive time points. Both WT and GLTP KO HeLa cells were seeded to poly-L-lysine-coated cell culture microplates (XF96; Agilent Technologies), at a density of 25,000 cells per well, and grown in DMEM medium. Prior to the experiment, the medium was changed to Seahorse XF DMEM pH 7.4 medium (Agilent Technologies) supplemented with 10 mM of glucose, 1 mM of pyruvate, and 2 mM of L-glutamine. After a 45 min incubation period in a non-CO_2_ incubator at 37 °C, the OCRs were measured using a Seahorse XF Cell Mito Stress Test kit (Agilent Technologies) with the following treatments: oligomycin (1.5 μM), FCCP (0.5 μM), and rotenone (0.5 μM) plus antimycin A (0.5 μM). Twelve 3-min cycles were measured for each condition and the OCR was normalized to the total cell number in each well determined by the CyQuant assay.

### Spheroid formation assay

To determine the effect of GLTP KO on the three-dimensional growth and spheroid-forming behavior of HeLa cells, a spheroid growth assay was carried out using WT and KO cells on Corning 96-well round-bottomed ultra-low-attachment plates (Corning Cat. No. 4515; Sigma-Aldrich). The assay was carried out according to the manufacturer’s protocol, with cells seeded at five concentrations (10,000, 5000, 1000, 200, and 40 cells per 100 μl per well) per cell line onto the plate. The growth of the spheroids under culture conditions was then documented over at least a 96-h period, with the spheroids imaged every 24 h. KO cells were also induced to express WT GLTP (GLTP rescue) or a GLTP mutant variant (ΔFFAT-GLTP) *via* transfection of 10 μg of vector plasmid through electroporation (Neon Transfection System; Thermo Fisher Scientific). This was carried out to determine whether the rescue or mutant protein expression altered the spheroid formation of KO1 and WT cells.

### Vesicle trafficking assay

The effect of GLTP KO on vesicular trafficking in the cells was studied using an assay with the tsO45 thermoreversible folding mutant of VSVG as part of a VSVG-GFP construct. tsO45 accumulates at the ERES at its nonpermissive temperature (canonically 39–40 °C (23, 24, 25)), due to its inability to incorporate into vesicles because of its temperature-induced misfolding. At its permissive temperature (canonically 30–32 °C (23, 24, 25)), tsO45 folds correctly and incorporates into vesicles. The addition of GFP to the construct allows observation and tracking of VSVG and VSVG-incorporating vesicles in cells *via* fluorescence microscopy. Any disruption or alteration of vesicle transport should therefore be observable *via* changes in the behavior of the fluorescently tagged vesicles.

Cells were transfected with 10 μg of the tsO45 VSVG-GFP construct vector plasmid *via* electroporation (Thermo Fisher Scientific Neon). The cells were thereafter given a 24-h recovery period, growing on glass cover slides in cell plates at the nonpermissive temperature of 40 °C. After the recovery period, the cell plates were transferred to the permissive temperature of 32 °C, with slides being retrieved and fixated for imaging (using 4% paraformaldehyde solution) at 60-min intervals from the time of the transfer until 120 min after the temperature shift, resulting in three timepoints (0 min, 60 min, and 120 min). The fixated slides were then mounted on object glasses and imaged and photographed under a fluorescence microscope (Zeiss Axio Vert.A1). Ordinarily, the VSVG-GFP construct accumulates and is retained at the ER and ERES at nonpermissive temperatures and is released from these sites at the permissive temperature; deviations from and/or delays in this process can be observed *via* fluorescence microscopy of the GFP tag, forming the foundation of the assay.

KO cells were also transfected (through electroporation) with mutant variants of GLTP together with the VSVG-GFP construct to determine the roles of specific interactions and functions of GLTP in affecting vesicle trafficking. A ΔFFAT-GLTP mutant, incapable of interacting with VAP-A (15), was used to characterize the role of GLTP-VAP interactions in vesicle trafficking, while a W96A-GLTP mutant, incapable of binding and transporting glycolipids (35), was used to determine whether GLTP’s functionality as a transport protein affected vesicle trafficking. WT GLTP was also transfected through electroporation into KO1 cells to validate the functionality of the mutant transfections and to act as a rescue assay for GLTP’s function. The WT-GLTP and mutant GLTP vector plasmids were cotransfected into KO1 cells together with the VSVG-GFP construct, after which the assay proceeded as described above.

### Immunofluorescence

To validate the intended function of the VSVG assay, the distribution of the VSVG-GFP construct in the cell was compared to immunofluorescent staining of organelles in the cell. At each aforementioned timepoint, the retrieved cells were fixated as mentioned above and then permeabilized (0,1% Triton-X100 solution), protein-blocked (5% bovine serum albumin solution), and stained for immunofluorescence using the relevant primary and secondary antibodies (α-SEC16A [rabbit, HPA005684; Sigma-Aldrich] for ERES primary staining and α-golgin-97 [mouse, A21270; Invitrogen] for Golgi apparatus primary staining; AlexaFluor 594 goat-anti-rabbit [A11012; Invitrogen] and goat-anti-mouse [A32742; Invitrogen] for respective secondary staining). The glass slides were thereafter mounted on object glasses and imaged and photographed under a fluorescence microscope.

### Statistical analysis of data

The data are presented as means ± SEMs for at least three independent experiments. The statistical significance was calculated using a Student’s *t* test for pairwise comparisons. The level of statistical significance was set at 0.05: ∗*p* ≤ 0.05, ∗∗*p* ≤ 0.01, and ∗∗∗*p* ≤ 0.005.

## Data availability

The datasets generated and analyzed during the current study are included in this published article and are available from the corresponding author on reasonable request. The datasets are available in the ArrayExpress Archive of Functional Genomics Data repository. Link https://www.ebi.ac.uk/arrayexpress/experiments/E-MTAB-12118 for experiment E-MTAB-12118.

## Supporting information

This article contains supporting information.

## Conflict of interest

The authors declare that they have no conflicts of interest with the contents of this article.
