# Supplementary material for: Glycolipid transfer protein knockout disrupts vesicle trafficking to the plasma membrane
Source: J Biol Chem. 2023 Mar 15;299(4):104607. doi: 10.1016/j.jbc.2023.104607 (PMC10140181; doi:10.1016/j.jbc.2023.104607)
Supplement: Supporting Information [file mmc1.docx]

**Glycolipid transfer protein knockout disrupts vesicle trafficking to the plasma membrane**

Henrik Nurmi, Anders P. E. Backman, Josefin Halin, Max Lönnfors, Tomas Blom*, Pia Roos-Mattjus, and Peter Mattjus.

Biochemistry, Faculty of Science and Engineering, Åbo Akademi University, Artillerigatan 6A, III, 20520 Turku, Finland

* Current address: Orion Pharma, P.O. Box 425, 20101 Turku, Finland

**Corresponding author** Peter Mattjus, peter.mattjus@abo.fi

**Supporting information.**

Figure S1. Schematic figure showing the GLTP locus in chromosome 12 as well as the sequencing data for the generated knockout cell lines.

Figure S2. The effects of GLTP knockout and GLTP mutants on HeLa cell phospholipid and GSL levels.

Figure S3 & S4. Visualization of VSVG-GFP in HeLa and GLTP mutant cells using 3D surface plot analysis.
