# Supplementary material for: Glycolipid transfer protein knockout disrupts vesicle trafficking to the plasma membrane
Source: J Biol Chem. 2023 Mar 15;299(4):104607. doi: 10.1016/j.jbc.2023.104607 (PMC10140181; doi:10.1016/j.jbc.2023.104607)
Supplement: Supporting Figure S1 [file mmc2.docx]

^GLTP chromosome 12 locus 12q24.11 reverse strand^


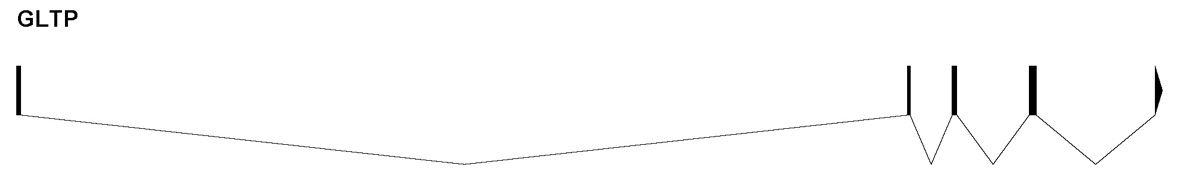

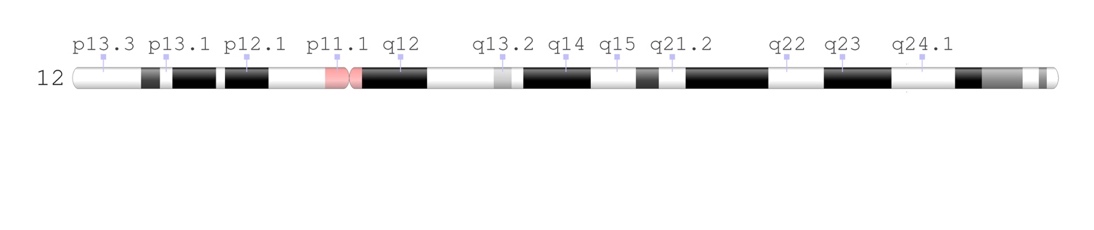


^exon 1 exon 2 exon 3 exon 4 exon 5^

^intron 1 intron 2 intron 3 intron 4^

^21530 bp 1023 bp 1756 bp 2881 bp^

^exon 1^

^guide A guide B^

M A L L A E H L L K P L P A D K Q I E T G P F L E A V S H L P P

GLTP atggcgctgctggccgaacacttgctgaagccgctgcccgcggacaagcagatcgagaccgggcccttcctcgaggcggtgtcccacctgccgccc

KO1^1^ atggcgctgctggcggtgtcc---------------------------------------------------------------cacctgccgccc

KO1^2^ atggcgctgctggccgaacacttgctgaagccgctgcacc------------------------------------------------tgccgccc

KO1^3^ atggcgctgctggcggtgtcccacctgccgcccttcttcggtgagccggaatggaggggggaggcggcccgcagccccacgcgcacccgaatcctc cttcccccgagcatcccctaaccatctctgccctagacacccctaagtactgtttccccaggcacccaagatcctctttccctccactcaacaagc

agatcgagaccgggccc

GLTP atggcgctgctggccgaacacttgctgaagccgctgcccgcggacaagcagatcgagaccgggcccttcctcgaggcggtgtcccacctgccgccc

KO2^1^ atggcgctgctggccgaacacttgg------------------acaagcagatcgagaccgggcccttcctcgaggcggtgtcccacctgccgccc

KO2^2^ atggcgctgctggccgaacacttg------------------------cggatcgagaccgggcccttcctcgaggcggtgtcccacctgccgccc

KO2^3^ atggcgctgctggccgaacacttgctgaagccgctgcccgcggacaagcagatcgggccgcggacaagcagatcgagaccgggcccttcctcgagg

cggtgtcccacctgccgccc

GLTP atggcgctgctggccgaacacttgctgaagccgctgcccgcggacaagcagatcgagaccgggcccttcctcgaggcggtgtcccacctgccgccc

KO3^1^ atggcgctgctggccgaacacttgctgaagccgctgc---------------------------------tcgaggcggtgtcccacctgccgccc

KO3^2^ atggtgtcccacc---------------------------------------------------------------------------tgccgccc

KO3^3^ atggcgctgctggccgaacacttgctgaagc---------------------------------cgttgctcgaggcggtgtcccacctgccgccc

GLTP target sense antisense

Exon1

Guide A CACCGCAGCGGCTTCAGCAAGTGTT AAACAACACTTGCTGAAGCCGCTGC

Guide B CACCGCGGACAAGCAGATCGAGACC AAACGGTCTCGATCTGCTTGTCCGC

**FIGURE S1. Schematic GLTP locus in chromosome 12 with GLTP gene overview and detailed view of exon 1 region.** Three GLTP KO clones (KO1, KO2 and KO3) were expanded and their genomic DNA extracted and sequenced. The resulting indels for each of the three alleles (numbered ^1-3^) of chromosome 12 are shown with nucleotide insertions indicated by red letters and deleted bases by dashed lines*.* The GLTP specific guide RNA primer sites are shown in yellow. Below the GLTP specific guide RNA primers used for sequencing.
