# Supplementary material for: Glycolipid transfer protein knockout disrupts vesicle trafficking to the plasma membrane
Source: J Biol Chem. 2023 Mar 15;299(4):104607. doi: 10.1016/j.jbc.2023.104607 (PMC10140181; doi:10.1016/j.jbc.2023.104607)
Supplement: Supporting Figure S2 [file mmc3.docx]

PI

PC

SM

std

WT1

WT2

KO1

KO2

KO2R

origin

KO1R

PE


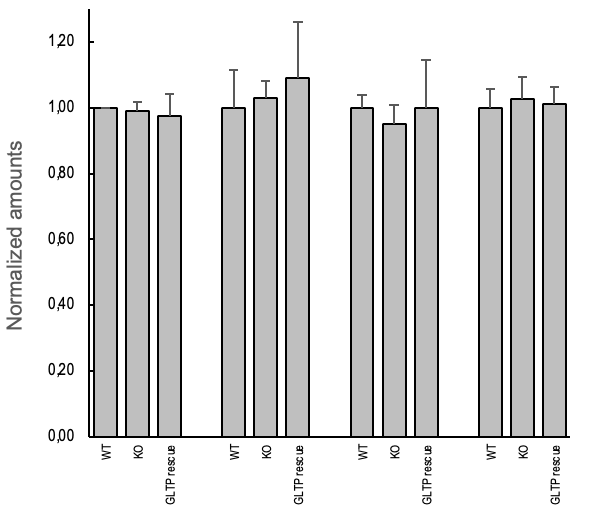


PI

PC

SM

PE


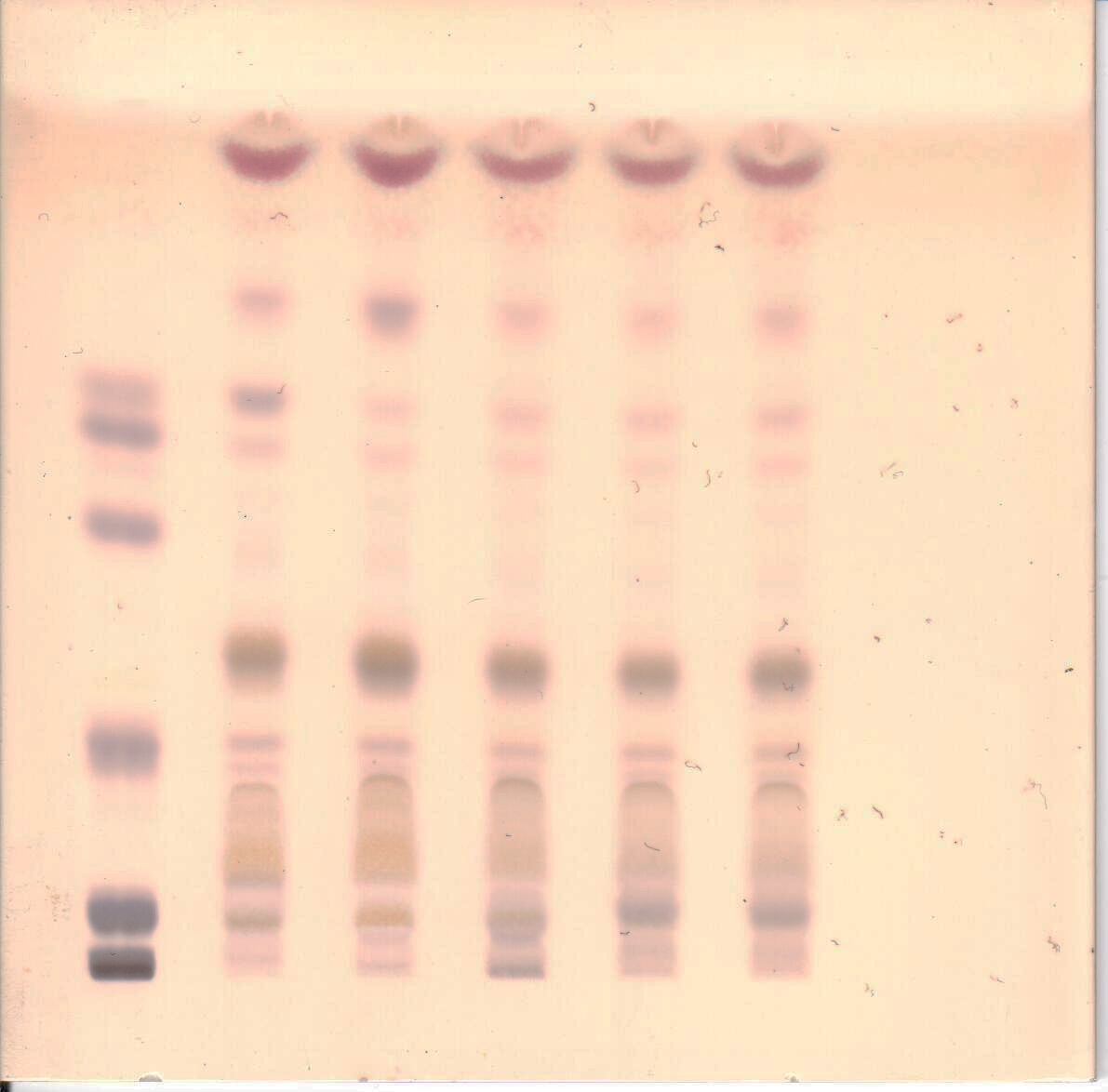


std

WT1

𝛥FFAT

KO1

KO1R

W96A

Cer

GlcCer

GalCer

OH-Gal-

Cer

LacCer

cGSL


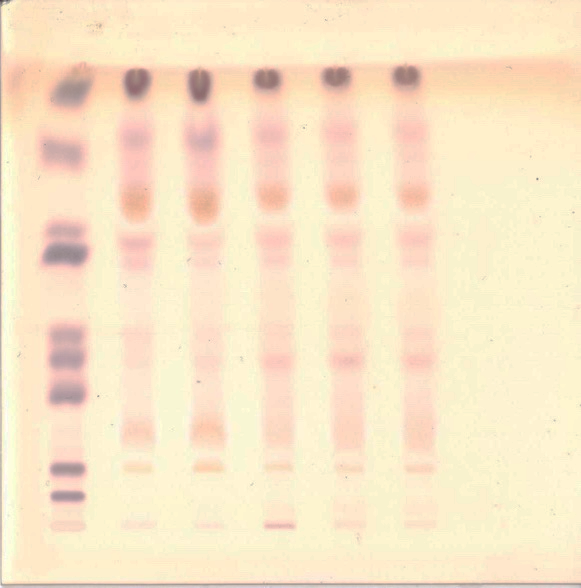


LacCer

Gb_3_

Gb_4_

GM_3_

GM_2_

origin

GM_1_

std

WT1

𝛥FFAT

KO1

KO1R

W96A

**FIGURE S2. Lipid levels in HeLa cells analyzed by HPTLC chromatography.** The upper section shows the HPTLC separation of phospholipids using the chloroform/methanol/acetic acid/water (25:15:4:2, v/v) solvent system and to the right normalized spot analysis using ImageJ. The lower left-hand HPTLC plate shows the separation of complex GSLs with the 25:20:4:1 chloroform:methanol:HAc:H_2_O (v/v) solvent system for WT, KO, KO rescue, the GLTP FFAT mutant and the GLTP W96A mutant introduced back to the GLTP KO cell line. The lower right-hand HPTLC plate shows the separation of GlcCer, GalCer, and LacCer using the solvent system 10:2:4:2:1 chloroform:methanol:acetone:HAc:H_2_O (v/v) for the same samples. The glycosphingolipids were visualized using orcinol and for the phospholipids, iodine or cupric acid. Std, lipid standards; WT, wild-type HeLa cells; KO, GLTP knockout; KOR, GLTP rescue; GLTP 𝛥FFAT.
