# Supplementary material for: Glycolipid transfer protein knockout disrupts vesicle trafficking to the plasma membrane
Source: J Biol Chem. 2023 Mar 15;299(4):104607. doi: 10.1016/j.jbc.2023.104607 (PMC10140181; doi:10.1016/j.jbc.2023.104607)
Supplement: Supporting Figure S3 [file mmc4.pdf]

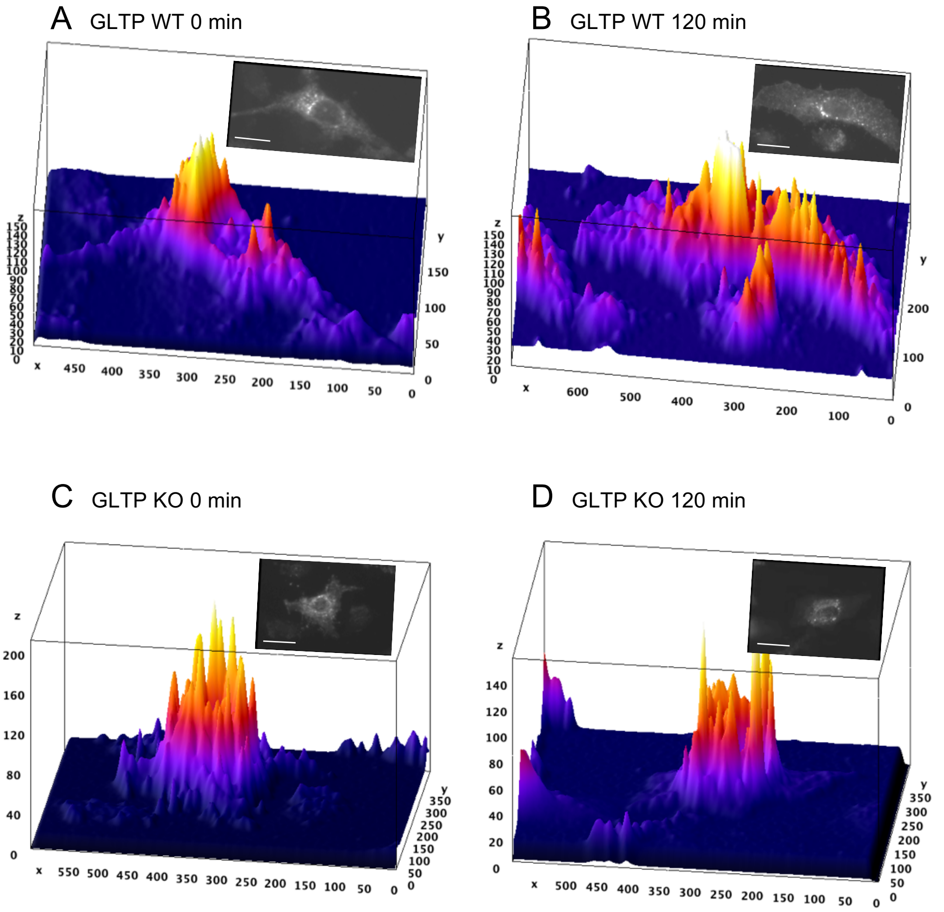

FIGURE S3. 3D surface plot of HeLa cells expressing VSVG-GFP.
We analysed the intensity of the GFP fluorescence cell images with the ImageJ software using the 3D interactive surface plot to further illustrate the intracellular localization of the VSVG-GFP construct. (A) & (B) Shows the WT HeLa cells expressing VSVG-GFP imaged at 0 (same cell as in Fig. 9A, rotated for a clearer 3D visualization) and 120 minutes (same cell as in Fig. 9C, rotated for a clearer 3D visualization). (C) & (D) Shows images of representative GLTP KO HeLa cells expressing VSVG-GFP imaged at 0 and 120 minutes (neighbouring cells from the same experiment as shown in Fig. 9D and 9F). Scale bars are 100 µm.
