# Supplementary material for: Glycolipid transfer protein knockout disrupts vesicle trafficking to the plasma membrane
Source: J Biol Chem. 2023 Mar 15;299(4):104607. doi: 10.1016/j.jbc.2023.104607 (PMC10140181; doi:10.1016/j.jbc.2023.104607)
Supplement: Supporting Figure S4 [file mmc5.pdf]

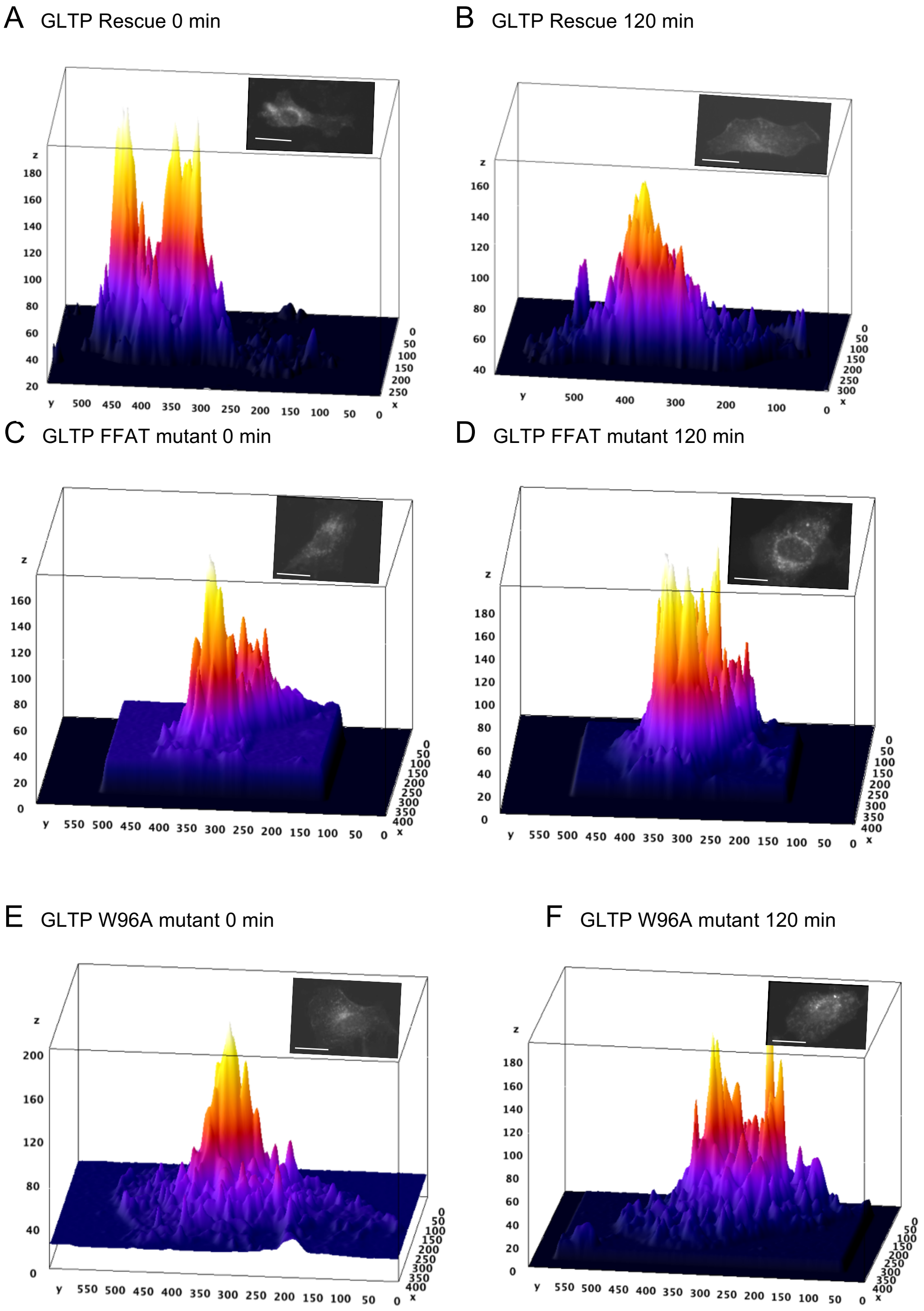

FIGURE S4. 3D surface plot of HeLa cells expressing VSVG-GFP.
We analysed the intensity of the GFP fluorescence cell images (inserts) with the ImageJ software using the 3D interactive surface plot. (A) & (B) Representative GLTP KO rescue HeLa cells expressing VSVG-GFP imaged at 0 and 120 minutes. (C) & (D) Representative GLTP FFAT mutant HeLa cells expressing VSVG-GFP imaged at 0 and 120 minutes. (E) & (F) Representative GLTP W96A mutant HeLa cells expressing VSVG-GFP imaged at 0 and 120 minutes. Scale bars are 100 µm.
